# Selective Preconcentration of Gold from Ore Samples

**DOI:** 10.1155/2018/7503202

**Published:** 2018-09-12

**Authors:** Hurmus Refiker, Melek Merdivan, Ruveyde Sezer Aygun

**Affiliations:** ^1^Department of Chemistry, Middle East Technical University, Ankara, Turkey; ^2^Institute of Applied Sciences, University of Kyrenia, Sehit Bakir Yahya Street, Kyrenia, North Cyprus, Mersin 10, Turkey; ^3^Department of Chemistry, Dokuz Eylul University, Izmir, Turkey

## Abstract

A simple and selective method has been developed for preconcentration of gold in ore samples. The method is based on use of N, N-diethyl-N'-benzoylthiourea (DEBT) as selective chelating agent and Amberlite XAD-16 as solid sorbent. Sorption behavior of gold with DEBT impregnated resin under optimized conditions has been studied in batch process. The gold ion capacity of the impregnated resin is calculated as 33.48 mg g^−1^ resin (0.17 mmol g^−1^ resin). The selective preconcentration of metal was examined using gold chelates prepared in column process under optimized conditions: pH, flow rate, volume of sample solution, nature of eluent, flow rate, and volume of eluent. Under optimum conditions, gold ions at the concentration of 0.015 *μ*g mL^−1^ with a preconcentration factor of 6.7 have been determined by flame atomic absorption spectrometry (FAAS). The accuracy of the proposed method was validated by the analysis of a Cu-ore (semi-certified) supplied by CMC (Cyprus Mining Company, North Cyprus) and a certified reference material, Gold Ore (MA-1b Canmet-MMSL). Satisfactory results were obtained with a RSD of 7.6%. The highly selective proposed method does not require any interference elimination process.

## 1. Introduction

Gold is one of the precious metals which occurs in very low natural contents such as 4 ng g^−1^ in basic rocks, 1 ng g^−1^ in soils, 0.05 *μ*g L^−1^ in sea water, and 0.2 *μ*g/L in river water. Due to its specific physical and chemical properties, gold is widely used in industry, agriculture, and medicine [1]. Low abundance and heterogeneous distribution of gold in geological samples and various interfering matrices requires the development of accurate and reliable analytical procedures for determination of gold in environmental samples. Therefore, a selective separation and preconcentration method is a critical need for sensitive, accurate, and interference free determination of gold [2].

In literature, various techniques have been recorded for separation and preconcentration of gold, such as liquid-liquid extraction [3], coprecipitation [4], solid-phase extraction [5], cloud point extraction [6], and electrodeposition [7]. Solid-phase extraction (SPE) is preferable over all these techniques due to its advantages like high enrichment factor, high recovery, rapid phase separation, low cost, minimum solvent waste generation, and sorption of the target species on the solid surface in a more stable chemical form [8, 9].

The use of solid sorbents for preconcentration and separation has received great attention from analytical chemists [10]. Among wide range of solid phases such as multiwalled carbon nanotubes [11], surfactant coated alumina [12], styrene-divinylbenzene matrix [5, 13, 14], and silica gel [10, 15] have gained much importance for the metal ion enrichment. Amberlite XAD series have been more preferably used as solid support due to their good physical properties such as high porosity, uniform pore size distribution, high surface area as chemical homogenous non-ionic structure, and good adsorbent properties for great amounts of uncharged compounds [5, 16, 17]. Compared to Amberlite XAD-2 and XAD-4 resins, Amberlite XAD-16 has larger surface area [9], which makes possible to increase the number chelating sites hence increasing the selectivity towards target metal ions. This can be achieved by selecting suitable chelating agents. The chelating groups that are widely used for preconcentration of precious metals are imidazole, thioguanidine, dithizone, mercapto groups, amino groups, and thioureas [18]. In several studies, N, N-diethyl-N'-benzoylthiourea (DEBT) has been recorded as a selective complexing agent for precious metals [19, 20]. Its selectivity is mainly controlled by pH. It has very high resistance to hydrolysis and oxidation. In addition to high pKs values, with resonance effects, DEBT can increase the electron density at sulfur donor atom when suitable acceptors are available. DEBT forms stable complexes only with Class b and border line acceptors. Noble metal ions, due to their specific Class b properties, form chelates with DEBT in low oxidation states in strongly acidic solutions [19, 20].

In the present study, DEBT as a chelating ligand and Amberlite XAD-16 as solid support have been used for selective separation and preconcentration of gold which is determined by flame atomic absorption spectrometry (FAAS). Optimum conditions for batch and column processes have been studied in detail. Then the proposed method has been applied to two real samples: Cu-ore supplied from CMC, North Cyprus, and a certified reference material Gold Ore (MA-1b) supplied by Canmet-MMSL, Ontario.

## 2. Experimental

### 2.1. Apparatus and Instrumentation

In order to prevent sorption of gold on silica surfaces, equipment made of polytetrafluoroethylene (PTFE) was used. 100 mL DuPont polyethylene containers for the storage and 5-50 and 100-1000 *μ*L adjustable micropipettes (Transferpette, Treff Lab) with disposable polyethylene tips for preparation of solutions were used. In batch process, optimum conditions such as pH, stirring time, metal ion capacity, and agents suitable for desorption were studied by using 50-mL of Falcon tubes. NÜVE SL 350 horizontal shaker was used during sorption optimizations in batch process.

Columns were prepared from 12-mL syringe barrels (1.5 cm i.d., 7.8 cm height, PTFE, Supelco) where disposable porous frits were placed at the bottom of the barrels. 1.0 g resin (unless otherwise stated) slurred in 50 mL water was uniformly placed in column and was covered with cotton wool to prevent dispersion by the addition of sample solution. Tygon® tubing was used to connect the outlet tip of the syringe barrel to a Gilson Miniplus peristaltic pump. A calibration, flow rate mL min^−1^ versus rpm was carried out. This calibration was repeated for each column before the application. Each time, 15 mL blank solutions at a flow rate of 1 mL min^−1^ were passed before sorption and desorption studies.

Philips PU 9200 Atomic Absorption Spectrometer with Epson FX-850 printer was used for determination of gold ions.

### 2.2. Chemicals

All the reagents were of analytical reagent grade. Deionized water from a Milli-Q system was used throughout the study unless otherwise stated. Amberlite XAD-16 resin was supplied by Sigma. Gold standard solutions were prepared by diluting of 1000 *μ*g mL^−1^ stock solution (Spectrosol) with 1 mol L^−1^ HCl (J.T. Baker, 36-38% w/w). During batch process, pH-adjustments were done using NaOH (Acros, 50% w/w). For desorption studies, Na_2_S_2_O_3_ (extra pure, Bilesik Kimya Mekanik) was used.

### 2.3. Synthesis of DEBT and Impregnation Process

DEBT was synthesized according to the procedure modified in our laboratory [20] where potassium thiocyanate (Fischer, 0.1mol) was dissolved in anhydrous acetone (Riedel-deHaen, 100 mL) by stirring and heating in a reflux condenser. After cooling to room temperature, benzoyl chloride (Merck, 0.1 mol) was added dropwise and stirred for 30 minutes. Then potassium salt was filtered off. The filtrate in orange was reacted with 0.1 mol of diethylamine (Merck) dropwise. The reaction mixture was crystallized in 250 mL of 1 mol L^−1^ HCl solution. After filtering the mixture, the residue was recrystallized with ethanol.

Since the impregnation process deals with physical interactions between the chelating agent and solid support by either inclusion in the pores of the support material or adhesion process or electrostatic interaction, some parameters controlling the impregnation such as stirring time and chelating agent capacity have been optimized as mentioned elsewhere [21].

### 2.4. Batch Method

With batch studies, sorption behavior of high concentrations of gold on DEBT impregnated Amberlite XAD-16 was investigated. Some critical parameters such as pH, stirring time, and metal ion capacity of resin capacity have been studied to find out the optimum conditions for recovery of gold.

#### 2.4.1. pH Effect

In order to investigate the pH effect on sorption of gold onto impregnated resin, different sets of 10 mL of 10 mg L^−1^ of Au^3+^ solution in the pH range of 1-5 were stirred with samples of 0.1 g impregnated resin for 50 minutes. After filtration under vacuum, metal ions in the filtrate were determined by FAAS.

#### 2.4.2. Stirring Time

Three different sets of 2 mg L^−1^, 10 mg L^−1^, and 100 mg^−1^L of 10 mL of Au^3+^ solutions in 1 mol L^−1^ HCl were stirred with samples of 0.1 g impregnated resin from the periods of 5 minutes to 1 hour. Then solutions were filtered and filtrates were aspirated into FAAS for metal ion determination.

#### 2.4.3. Gold Ion Capacity of Resin

In order to determine the resin capacity, samples of 0.1 g of impregnated resin (1 mmol DEBT g^−1^ resin) were stirred with 10 mL of gold ions solutions in the concentration range of 2 mg L^−1^ to 600 mg L^−1^ in 1 mol /L^−1^ HCl for 15 minutes. Then the solutions were filtered and metal ion concentrations were determined by FAAS.

### 2.5. Column Method

Since the kinetic and equilibrium aspects of column process are different than batch process, optimization of column conditions is needed. Effect of flow rate and volume of ligand solution on impregnation and effect of ligand concentration on amount of metal chelate adsorption have been studied in column process.

### 2.6. Proposed Method for Preconcentration of Gold

100 mL of gold chelate solutions (0.15 *μ*g mL^−1^ Au^3+^ and 3 mL of 2x10^−3^ mol L^−1^ DEBT) was percolated through the column (1.0 g pure resin) at a flow rate of 0.5 mL min^−1^. Then metal ions could be eluted with 15 mL of 0.2 mol L^−1^ Na_2_S_2_O_3_ in water with a recovery of 97.6 ± 2.3% (N=2).

### 2.7. Preparation of Ore Samples

An acid digestion procedure was applied to Cu-ore and Gold Ore (MA-1b) samples as suggested elsewhere [22]. Accordingly, two parallel 10.0 g of Cu-ore sample and 1.0 g of Gold Ore (MA-1b) were transferred into Teflon beakers. 20 mL of HCl was added to each where the beakers were covered and placed on a warm hot plate. After 15 minutes of digestion, 15 mL of concentrated nitric acid was added and the contents were digested for 20 minutes. Then 25 mL of concentrated HCl and 25 mL of deionized water were added. The contents were boiled to expel nitric acid digestion gases and to dissolve all soluble salts. After cooling they were filtered through Whatman white band filter paper into 100 ml PTFE flask. Once 3 mL of 2 X 10^−3^ mol L^−1^ DEBT solution was added, final volume was completed to 100 mL with deionized water. Later, the proposed procedure for preconcentration of gold was applied.

## 3. Results and Discussion

### 3.1. Characterization Studies

The structure of DEBT was characterized by FTIR and UV-VIS spectrophotometer. The synthesized DEBT exhibited 2 strong broad UV-absorption peaks at 237 and 278 nm which were consistent with those given in literature [20]. The characteristic absorption bands for N-H, C−H, and amide I (C=O), amide II, and amide III at 3276, 3066-2936, 1656, 1537, and 1306 cm^−1^, respectively, appeared in both of the spectra. FTIR studies for pure resin, DEBT, DEBT impregnated resin, and DEBT-metal chelates have been carried out [20]. The characteristic absorption bands for C-H and amide I (C=O), amide II, and amide III at 3276, 3066-2936, 1656, 1537, and 1306 cm^−1^, respectively, appeared only in the spectra of DEBT and impregnated resin. While the characteristic IR band of –N(CH_2_CH_3_)_2_ group in the ligand at 2875 cm^−1^ remained almost unchanged in the spectrum of the complex showing that this group is not involved in coordination, C-H vibration in the aromatic ring is blue shifted upon metal-ligand bond formation. The position of amide I, amide II, and III bands at 1656, 1537, and 1306 cm^−1^, respectively, arising from the carbonyl of the benzamide moiety and secondary amide of DEBT at 3276 cm^−1^ disappeared in the complex.

### 3.2. Parameters Optimized in Batch Method

#### 3.2.1. pH Effect

In literature, it is noted that DEBT forms stable and selective complexes with noble metals only in acidic or strongly acidic media [19]. Moreover, Shuster and coworker reported optimum pH range as 0-5 for liquid-liquid extraction of gold with DEBT was previously reported as 0-5 by Schuster and coworkers [31]. Therefore, the pH effect on chelation of gold ions with DEBT impregnated resin is investigated within the pH range from 1 to 5.

**Figure 1:**
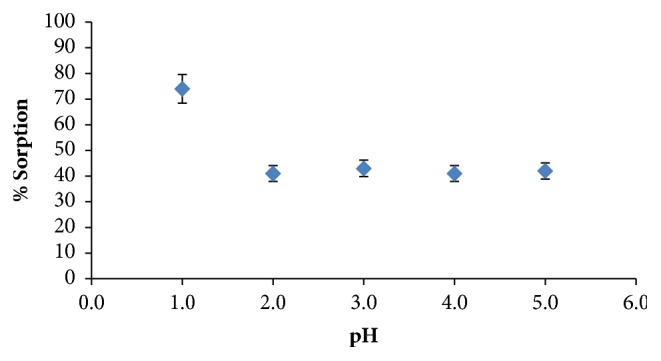
Effect of pH on sorption. Amount of resin: 0.1 g resin, amount of DEBT: 1mmol g^−1^ resin, and stirring time: 50 minutes.

It was shown that the maximum percent sorption is obtained at pH ~ 1 (see Figure 1). Therefore, standard solutions were prepared by diluting AAS standard stock solutions with 1 mol L^−1^ HCl.

#### 3.2.2. Effect of Stirring Time

**Figure 2:**
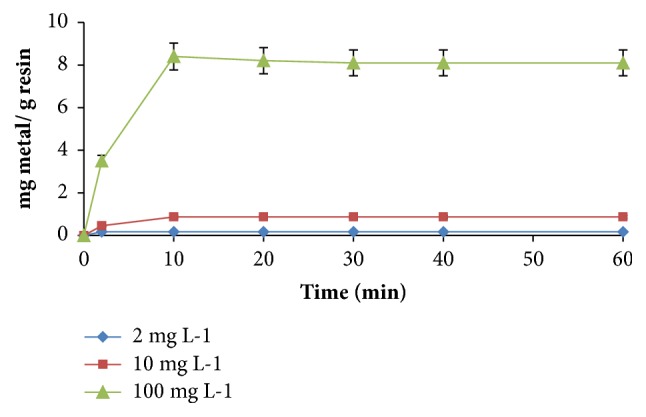
Stirring time of gold. Amount of resin: 0.1 g and amount of DEBT: 1 mmol g^−1^ resin.

Referring to Figure 2, it can be concluded that 15 minutes of stirring is sufficiently good enough to achieve sorption equilibrium for three different concentrations of gold ion. Higher gold ion concentrations have no effect on the optimum time of sorption. Actually, fast kinetics can be expected in applications of macroporous resins. In addition, high ligand concentration, 1 mmol g^−1^ resin, used in impregnation may increase selectivity of the resin which can also be a reason for fast sorption rate.

As a result, 15 minutes of stirring can be accepted as a suitable stirring time during loading of resin with possible higher concentrations of gold ions to determine gold ion capacity of the resin.

#### 3.2.3. Gold Ion Capacity of Impregnated Resin

**Figure 3:**
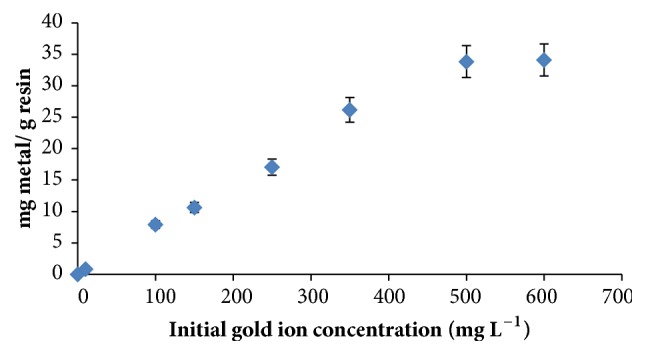
Gold ion capacity of resin. Amount of resin: 0.1 g, amount of DEBT: 1 mmol g^−1^ resin, and stirring time: 15 minutes.

Referring to Figure 3, after 500 mg L^−1^ of gold ions solutions, the impregnated resin reaches to saturation. The metal ion capacity of the resin is calculated as 33.48 mg Au^3+^ g^−1^ resin (0.17 mmol Au^3+^ g^−1^ resin) applying the following formula [32].

(1) $$\begin{array}{c} Q=\frac{\left(C_{o}-C_{A}\right)\times V}{W} \end{array}$$

where Q is the metal ion capacity (mg/g),

 
C_o_ is the initial concentration of metal ion (mg/L),
 
C_A_ is the equilibrium concentration of metal ion (mg/L),
  V is the volume of the solution (L),
  W is the weight of the resin (g).

### 3.3. Optimized Parameters in Column Method

During the application of the proposed method in column process, it was noticed that excess volume of sample solutions during sorption leached the impregnated DEBT that lead to loss of selectivity and analyte. Moreover, the partial exhaustion of available chelating sites due to leaching of impregnated ligand caused irreproducible results of sorption percentages of metal ion [5]. Therefore, research was continued with preparation of metal chelates before transferring to column and certain limited volume of chelate solution would be percolated through the column containing pure resin under the optimized conditions.

#### 3.3.1. Sample Flow Rate of Gold Chelates

During batch studies, it was recognized that DEBT showed similar kinetics during chelation with gold as that of silver which was reported in the previous study [5], as long as DEBT concentration is kept the same or close optimum pH for sorption is maintained [21]. In the previous study, considering application of larger volume of sample solutions for preconcentration, to be on safe 0.5 mL min^−1^ had been accepted as optimum sample flow rate [5]. The same was also found to be optimum sample flow rate for gold studies.

#### 3.3.2. Effect of Ligand Volume on Impregnation of Gold Chelates onto Resin

**Figure 4:**
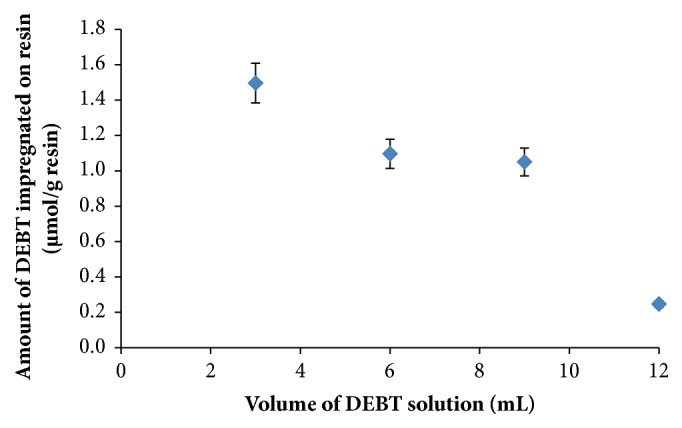
Effect of ligand volume on impregnation. Initial DEBT-ethanol concentration: 3.75 x 10^−4^ mol L^−1^ and flow rate: 0.5 mL min^−1^.

Maximum applicable ligand volume and concentration on analyte sorption are important. Therefore, maximum applicable ligand volume on retention is studied before further application of metal chelates for solid-phase extraction. For this reason, 3 mL of 3.75x10^−4^ mol L^−1^ DEBT solution was percolated through column for 4 times and DEBT concentration in the effluent determined by UV spectrometry. In Figure 4, it can be seen that maximum amount of DEBT retained on Amberlite XAD-16 was achieved with the first 3 mL of DEBT solution. Following additions of 3 mL of 3.75x10^−4^ mol L^−1^ DEBT solution showed a decrease in amount of DEBT retained on resin. This may be because of the leaching effect of ethanol on DEBT.

#### 3.3.3. Effect of Optimized Ligand Concentration on Amount of Gold Chelates

Ligand concentration is also important because if it is not excessively present, the chelate formation may not be complete so metal ions may not be selectively retained on resin. However, excess DEBT (in case of inadequate amount of resin) may prevent retention of metal chelates because of the competition for sorption on resin between excess DEBT and metal chelates.

Considering the further applications of the proposed method to a real sample and limitations related to ligand mentioned above, it was decided to use 1.0 g of resin and amount of DEBT as 3 mL of 2x10^−3^ mol L^−1^. This amount of DEBT is always in excess considering the amounts of analyte metal that is our concern (15 *μ*g gold ions).

As indicated in Table 1, up to 100 *μ*g gold ions in 10 mL sample solution can be safely retained on 1.0 g of resin as metal chelates. When 10 mL of 50 *μ*g mL^−1^ metal chelate solution was passed through the column, a decrease in percent retention was observed.

During this study, we dealt with quite low amounts of metal ions and carried out the optimizations accordingly. Referring to results in Table 1, any researchers interested in higher amounts of gold up to 100 *μ*g can study safely with the proposed method under the same conditions (such as amount of resin, sample flow rate, pH of sorption media, and ligand volume) as long as only the concentration of eluent and its volume are reoptimized according to the interested amount of metal ions.

#### 3.3.4. Choice of Eluent: Its Nature, Concentration, Volume, and Flow Rate

In the previous study, we used sodium thiosulfate which was found as the most suitable eluent for desorbing silver ions from Amberlite XAD-16 [5]. The sorption of silver ions was governed by the chelation mechanism in that silver ions (belong to class of soft acids) have affinity for (S-O) chelating group of DEBT. During desorption, (S-S) chelating groups of Na_2_S_2_O_3_ provide a stronger complex formation as silver has a higher affinity for (S-S) than (S-O) chelating group [21]. Since gold metal also belongs to class of soft acid and same discussion could be valid as well, as a result, Na_2_S_2_O_3_ was selected as suitable eluent for desorbing gold ions.

Series of experiments were conducted to optimize the concentration and the volume of eluent [21]. When 0.1 mol L^−1^ Na_2_S_2_O_3_ in water was used as an eluent, at a flow rate of 0.3 mL min^−1^, only 65% desorption was obtained. Later, it was found that the highest recovery with 97.6 ± 2.3% desorption was achieved when 15 mL of 0.2 mol L^−1^ Na_2_S_2_O_3_ in water was percolated through the column at a flow rate of 0.3 mL min^−1^.

### 3.4. Effect of Electrolytes and Competing Ions

In geological samples like ores, some metals in higher concentrations such as Na^+^, K^+^, Cu^2+^, Ni^2+^, Pb^2+^, Mn^2+^, Fe^3+^, Zn^2+^, Al^3+^, and Cr^3+^ can coexist with gold. The anions Cl^−^, NO_3_^−^, SO_4_^2−^, PO_4_^3−^, and ClO_4_^−^ are the anions that are capable of forming complexes with several metal ions.

Considering the real sample amount weighed according to gold digestion procedure (at least 10.0 g), 50 mL of 300 *μ*g mL^−1^ of copper standard solution was prepared in 1 mol L^−1^ HCl. Initial metal ion concentration was determined by FAAS. Then the proposed method was applied. The metal ion concentration in the effluent was determined by FAAS. Initial metal ion concentration was found to be 292 *μ*g mL^−1^ and the metal ion concentration in the effluent was found as 288 *μ*g mL^−1^. As a result, only 1.37% of copper ions was adsorbed on resin. Although it is known that copper ion forms complex with DEBT in pH range of 0-7 [20], this result showed that the formation of Cu-DEBT complex in 1 mol L^−1^ HCl is quite lower to compete with gold ions. N-benzoylthioureas are bidentate chelating ligands with S and O as donor atoms. The possibility of increasing the electron density at the sulfur atom by means of resonance effect leads to selective complex behavior of DEBT which can be influenced by the adjustment of pH, where competing metal ions could be eliminated.

The effect of various electrolytes like NaNO_3_, Na_2_SO_4_, Na_3_PO_4_, and Na_2_CO_3_ on the sorption of gold (1 mg L^−1^) as Au-DEBT chelate on Amberlite XAD-16 resin was studied as well. Na_2_SO_4_ was tolerable up to 0.04 mol L^−1^, Na_3_PO_4_ up to 0.1 mol L^−1^, and NaNO_3_ and Na_2_CO_3_ up to 0.15 mol L^−1^.

### 3.5. Analytical Figures of Merit

The calibration graph for the determination of gold was plotted according to the proposed procedure under the optimum conditions. The equation of the line was derived as A = 0.0125C + 0.0003 with the regression coefficient 0.9998 where A is the absorbance and C is concentration of the metal ion (*μ*g mL^−1^).

The limit of detection (LOD) and limit of quantitation (LOQ) for gold ions were determined employing the standard solutions giving absorbance signal slightly recognizable than blank. The LOD and LOQ were calculated based on 3s/slope and 10s/slope of 10 measurements of the blank, respectively, where s is the standard deviation of the sample solution. The results of the LOD, LOQ, and precision (RSD %) for gold and its concentration are shown in Table 2.

### 3.6. Analysis of Real Samples

In order to demonstrate the accuracy of the proposed method, analyses of two real samples, one of which is the Cu-ore supplied by Cyprus Mining Company (CMC), North Cyprus, and the other one certified Gold Ore (MA-1b) by Canmet, Ontario, were carried out and the results were compared with the values reported. The results of spiked CMC samples and the results that were corrected for 97% desorption recovery value which was found for 100 mL sample solution were also tabulated in Table 3. Student's t-test was performed to statistically evaluate the found and certified values. The found values were in good agreement with the certified ones and the difference was found to be statistically insignificant (at 95% confidence interval level).

## 4. Conclusions

Highly selective, reliable, and low cost method has been proposed for preconcentration of gold ions from highly interfering matrices, namely ores. The validity of the proposed method was demonstrated by the analyses of two geological samples: Cu-ore (supplied by CMC) and Gold Ore (MA-1b) as a certified reference material. The results are in good agreement with the given values. Comparison of the proposed method with some similar studies in literature is summarized in Table 4. Although there are more sensitive methods applied to similar samples, such as ICP-MS and GFAAS, these are much more expensive and sophisticated. The gold ion at such a low concentration of 0.15 *μ*g mL^−1^ could be preconcentrated selectively and determined by the proposed method without any matrix elimination processes.

## Figures and Tables

**Table 1:** Effect of ligand concentration of retention of metal chelates.

Amount of DEBT	Amount of Au^3+^ in sample solution (*μ*g)	% Sorption of gold chelates on resin
3mL of 2x10^−3^ mol L^−1^	15	100 ± 2
3mL of 2x10^−3^ mol L^−1^	100	100 ± 2
3mL of 2x10^−3^ mol L^−1^	500	94 ± 3

Amount of resin: 1.0 g, sample volume: 10 mL, and sample flow rate: 0.5 mL min^−1^.

**Table 2:** Analytical figures of merit.

Initial concentration of solution^a^	Regression equation^b^	R^2^	LOD^c^	LOQ^d^	%RSD^e^	PF^f^
			(*μ*g mL^−1^)	(*μ*g mL^−1^)		
0.15 *μ*g mL^−1^	A = 0.0125C + 0.0003	0.9998	0.025	0.085	7.56	6.7

^a^sample volume = 100 mL, ^b^A(absorbance) = slope x C(concentration *μ*g mL^−1^) + intercept, ^c^limit of detection (2.5 *μ*g g^−1^ ore), ^d^limit of quantitation (8.5 *μ*g g^−1^ ore), ^e^percentage relative standard deviation, and ^f^preconcentration factor.

**Table 3:** Determination of Au in CMC ore sample and Gold Ore (MA-1b) CRM.

Samples	Au concentration	Corrected values according to 97% desorption
	(mgkg^−1^)	
CMC sample		
Au (found) *∗*	< LOD	< LOD
Au (claimed)	1.34	1.34
Au (spiked found) *∗*	13.6 ± 0.6	14.1 ± 0.6
Au (spiked)	15	15
Gold Ore (MA-1b)		
Au (found) *∗*	15.0 ± 1.0	15.5 ± 1.0
Au (certified value)	17.0 ± 0.3	17.0 ± 0.3

*∗* Values are given as mean ± SD, N = 3 (number of replicates).

**Table 4:** Comparison of the proposed method with some studies based on SPE and determination of gold reported in literature.

Adsorbent	Medium	Eluent	D. M.	LOD	Matrix	Ref.
Octadecyl silica membrane discs modified with pentathia-15-crown-5	pH 4.5-7.00	0.5 mol L^−1^ Sodium thiosulphate	FAAS	1.0 *μ*g L^−1^	Pharmaceutical and water samples	[23]

Diethyldithiocarbamate complex on Amberlite XAD-2000	0.5-2.5 mol L^−1^ HNO_3_	1 mol L^−1^ HNO_3_ in acetone	FAAS	16.6 *μ*g	Environmental samples	[14]

1-phenyl-1,2-propanedione-2-complex on oximethiosemicarbazone SP Sephadex C25	pH 3	__	ICP-MS	1.6X10^−8^-141X10^−8^ mol L^−1^	Minerals and natural water samples	[24]

Poly(N-(hydroxymethyl)methacylamide 0-1-allyl-thiourea) hydrogels	pH 0.5	0.8 mol L^−1^ thioura in 3 mol L^−1^ HCl	GFAAF	3 ng L^−1^	Anode slime and geological samples*∗*	[25]

Dowex M 4195 chelating resin	pH 4	2 mol L^−1^ H_2_SO_4_ + 2 mol L^−1^ NH_3_	FAAS	1.61 *μ*g L^−1^	Water, soil and sediment samples	[26]

Multi-walled carbon nanotubes	pH 1-6	3% thiourea in 1 mol L^−1^ HCl	FAAS	0.15 *μ*g L^−1^	Geological and water samples	[11]

2-pyridine-5-(4-tolyl)-1,3,4-oxadiazole complex on Amberlite XAD-4	0.5 mol/L HNO_3_	1 mol/L HCl in acetone	FAAS	1.03 *μ*g L^−1^	Environmental Samples	[27]

Polyethylenimine coated on Al_2_O_3_	pH 5.7	0.5 mol L^−1^ thiourea then 1.0 mol/L HCl	FAAS	26.2 ng L^−1^	Water Samples	[28]

Rubeanic acid complex on silica gel	pH 3.5	0.5 mol L^−1^ thiourea then 1.0 mol L^−1^ HCl	FAAS	0.80 ng mL^−1^	Water Samples	[29]

Silica gel (SG-CIPrNTf_2_)	pH 2	__	ICP-OES	__	Water Samples	[30]

DEBT complex on Amberlite XAD-16	pH~1	0.2 mol L^−1^ sodium thiosulphate	FAAS	0.025 *μ*g mL^−1^	Cu and Au Ores	This study

D.M.: detection method, Ref.: references. *∗*Matrix elimination method is used.

## Data Availability

No data were used to support this study.
